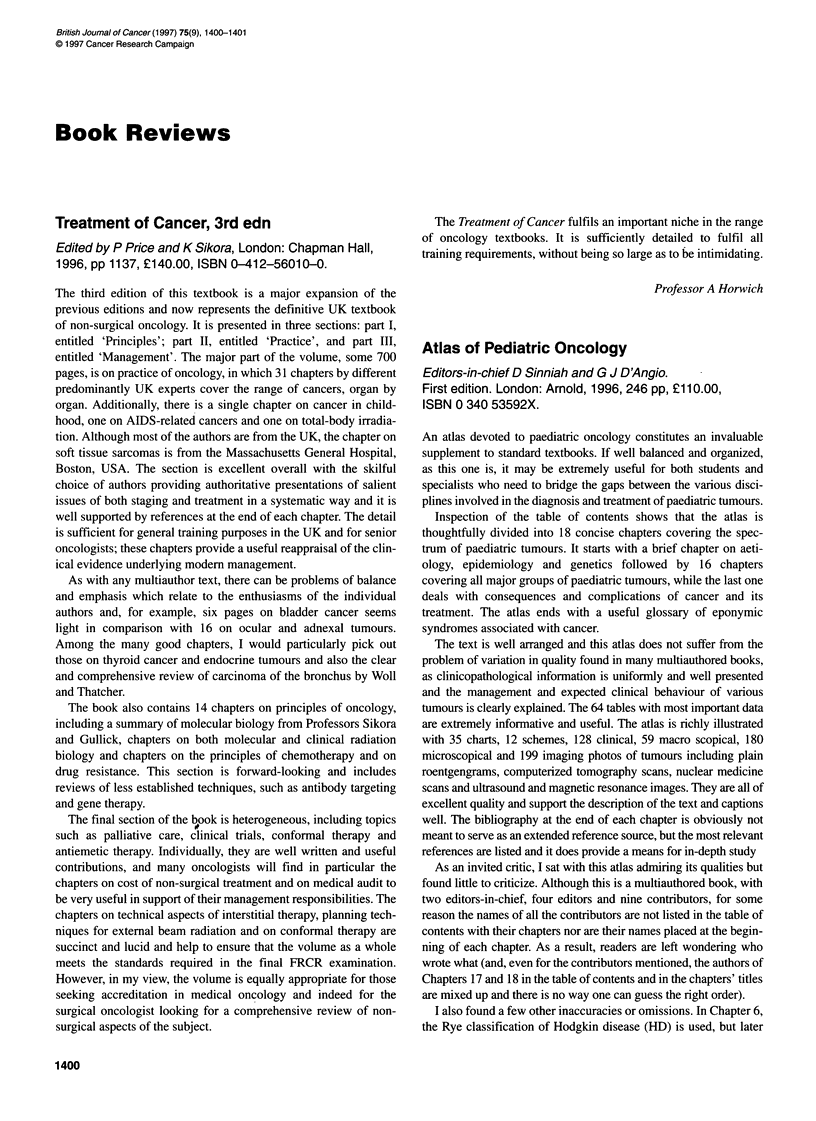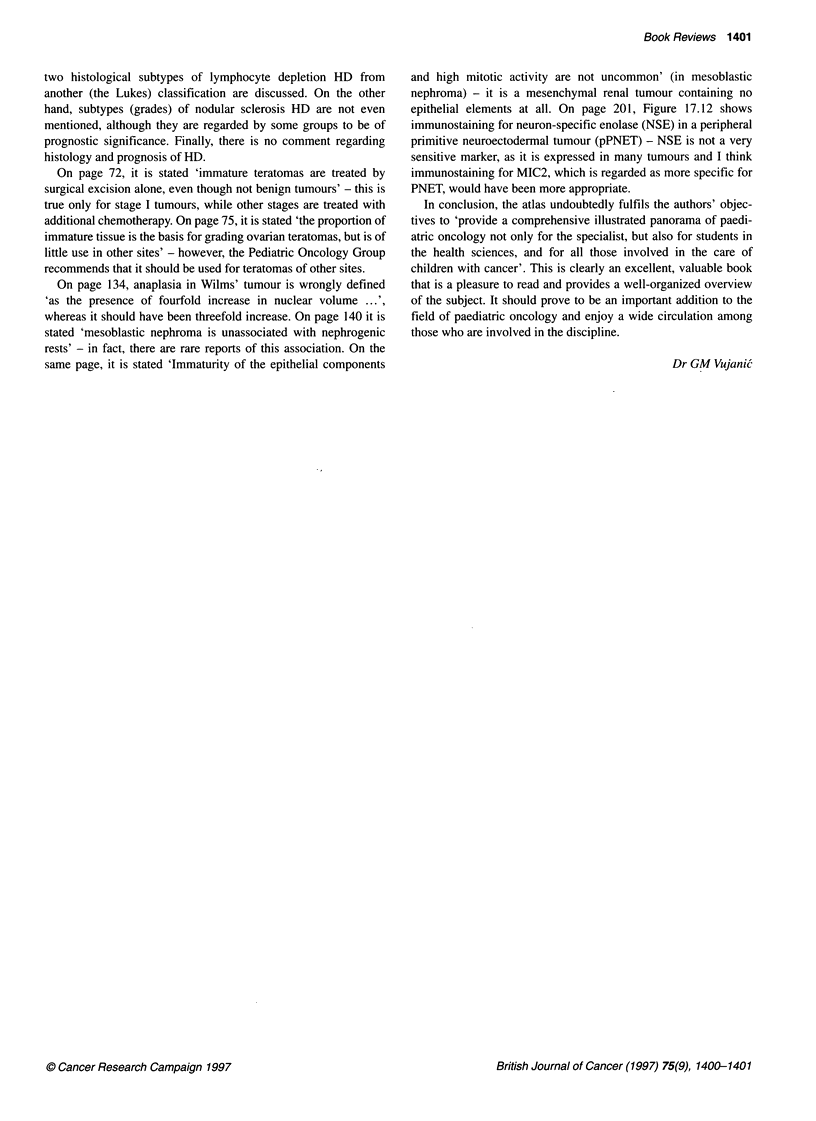# Atlas of Pediatric Oncology

**Published:** 1997

**Authors:** GM Vujanić


### Atlas of Pediatric Oncology

*Editors-in-chief D Sinniah and G J D'Angio.*
First edition. London: Arnold, 1996, 246 pp, £110.00, ISBN 0 340 53592X.

An atlas devoted to paediatric oncology constitutes an invaluable supplement to standard textbooks. If well balanced and organized, as this one is, it may be extremely useful for both students and specialists who need to bridge the gaps between the various disciplines involved in the diagnosis and treatment of paediatric tumours.

Inspection of the table of contents shows that the atlas is thoughtfully divided into 18 concise chapters covering the spectrum of paediatric tumours. It starts with a brief chapter on aetiology, epidemiology and genetics followed by 16 chapters covering all major groups of paediatric tumours, while the last one deals with consequences and complications of cancer and its treatment. The atlas ends with a useful glossary of eponymic syndromes associated with cancer.

The text is well arranged and this atlas does not suffer from the problem of variation in quality found in many multiauthored books, as clinicopathological information is uniformly and well presented and the management and expected clinical behaviour of various tumours is clearly explained. The 64 tables with most important data are extremely informative and useful. The atlas is richly illustrated with 35 charts, 12 schemes, 128 clinical, 59 macro scopical, 180 microscopical and 199 imaging photos of tumours including plain roentgengrams, computerized tomography scans, nuclear medicine scans and ultrasound and magnetic resonance images. They are all of excellent quality and support the description of the text and captions well. The bibliography at the end of each chapter is obviously not meant to serve as an extended reference source, but the most relevant references are listed and it does provide a means for in-depth study

As an invited critic, I sat with this atlas admiring its qualities but found little to criticize. Although this is a multiauthored book, with two editors-in-chief, four editors and nine contributors, for some reason the names of all the contributors are not listed in the table of contents with their chapters nor are their names placed at the beginning of each chapter. As a result, readers are left wondering who wrote what (and, even for the contributors mentioned, the authors of Chapters 17 and 18 in the table of contents and in the chapters' titles are mixed up and there is no way one can guess the right order).

I also found a few other inaccuracies or omissions. In Chapter 6, the Rye classification of Hodgkin disease (HD) is used, but later two histological subtypes of lymphocyte depletion HD from another (the Lukes) classification are discussed. On the other hand, subtypes (grades) of nodular sclerosis HD are not even mentioned, although they are regarded by some groups to be of prognostic significance. Finally, there is no comment regarding histology and prognosis of HD.

On page 72, it is stated 'immature teratomas are treated by surgical excision alone, even though not benign tumours' – this is true only for stage I tumours, while other stages are treated with additional chemotherapy. On page 75, it is stated 'the proportion of immature tissue is the basis for grading ovarian teratomas, but is of little use in other sites' – however, the Pediatric Oncology Group recommends that it should be used for teratomas of other sites.

On page 134, anaplasia in Wilms' tumour is wrongly defined 'as the presence of fourfold increase in nuclear volume ...', whereas it should have been threefold increase. On page 140 it is stated 'mesoblastic nephroma is unassociated with nephrogenic rests' – in fact, there are rare reports of this association. On the same page, it is stated 'Immaturity of the epithelial components and high mitotic activity are not uncommon' (in mesoblastic nephroma) – it is a mesenchymal renal tumour containing no epithelial elements at all. On page 201, Figure 17.12 shows immunostaining for neuron-specific enolase (NSE) in a peripheral primitive neuroectodermal tumour (pPNET) – NSE is not a very sensitive marker, as it is expressed in many tumours and I think immunostaining for MIC2, which is regarded as more specific for PNET, would have been more appropriate.

In conclusion, the atlas undoubtedly fulfils the authors' objectives to 'provide a comprehensive illustrated panorama of paediatric oncology not only for the specialist, but also for students in the health sciences, and for all those involved in the care of children with cancer'. This is clearly an excellent, valuable book that is a pleasure to read and provides a well-organized overview of the subject. It should prove to be an important addition to the field of paediatric oncology and enjoy a wide circulation among those who are involved in the discipline.

*Dr GM Vujanić*

© Cancer Research Campaign 1997